# The TAXINOMISIS Project: A multidisciplinary approach for the development of a new risk stratification model for patients with asymptomatic carotid artery stenosis

**DOI:** 10.1111/eci.13411

**Published:** 2020-10-02

**Authors:** Nathalie Timmerman, George Galyfos, Fragiska Sigala, Kalliopi Thanopoulou, Gert J. de Borst, Lazar Davidovic, Hans‐Henning Eckstein, Nenad Filipovic, Roberto Grugni, Michael Kallmayer, Dominique P. V. de Kleijn, Igor Koncar, Michalis D. Mantzaris, Elisabeth Marchal, Miltiadis Matsagkas, Perica Mutavdzic, Domenico Palombo, Gerard Pasterkamp, Vassiliki T. Potsika, Evangelos Andreakos, Dimitrios I. Fotiadis, all partners of the TAXINOMISIS Consortium

**Affiliations:** ^1^ Department of Vascular Surgery Division of Surgical Specialties University Medical Centre Utrecht Utrecht University Utrecht the Netherlands; ^2^ First Propedeutic Department of Surgery National and Kapodistrian University of Athens Athens Greece; ^3^ Laboratory of Immunobiology Center for Clinical Experimental Surgery and Translational Research Biomedical Research Foundation of the Academy of Athens Athens Greece; ^4^ Clinic for Vascular and Endovascular Surgery Serbian Clinical Center Belgrade Serbia; ^5^ School of Medicine University of Belgrade Belgrade Serbia; ^6^ Clinic and Policlinik for vascular and endovascular Surgery Klinikum rechts der Isar Technical University Munich Munich Germany; ^7^ BioIRC, Research and Development Center for Bioengieering Kragujevac Serbia; ^8^ Faculty of Engineering University of Kragujevac Kragujevac Serbia; ^9^ Engineering Ingegneria Informatica Milan Italy; ^10^ Department of Materials Science and Engineering Unit of Medical Technology and Intelligent Information Systems University of Ioannina Ioannina Greece; ^11^ Department of Life Science Technologies Imec Leuven Belgium; ^12^ Department of Vascular Surgery Faculty of Medicine University of Thessaly Thessaly Greece; ^13^ Division of Vascular and Endovascular Surgery IRCCS Ospedale Policlinico San Martino Genoa – Italian Cardiovascular Network Genoa Italy; ^14^ Division Laboratories and Pharmacy Laboratory of Clinical Chemistry and Hematology University Medical Centre Utrecht Utrecht University Utrecht the Netherlands; ^15^ Institute of Molecular Biology and Biotechnology Foundation for Research and Technology‐Hellas (FORTH) Ioannina Greece

**Keywords:** biomarker, carotid artery stenosis, pharmacogenetics, risk stratification

## Abstract

**Introduction:**

Asymptomatic carotid artery stenosis (ACAS) may cause future stroke and therefore patients with ACAS require best medical treatment. Patients at high risk for stroke may opt for additional revascularization (either surgery or stenting) but the future stroke risk should outweigh the risk for peri/post‐operative stroke/death. Current risk stratification for patients with ACAS is largely based on outdated randomized‐controlled trials that lack the integration of improved medical therapies and risk factor control. Furthermore, recent circulating and imaging biomarkers for stroke have never been included in a risk stratification model. The TAXINOMISIS Project aims to develop a new risk stratification model for cerebrovascular complications in patients with ACAS and this will be tested through a prospective observational multicentre clinical trial performed in six major European vascular surgery centres.

**Methods and analysis:**

The risk stratification model will compromise clinical, circulating, plaque and imaging biomarkers. The prospective multicentre observational study will include 300 patients with 50%‐99% ACAS. The primary endpoint is the three‐year incidence of cerebrovascular complications. Biomarkers will be retrieved from plasma samples, brain MRI, carotid MRA and duplex ultrasound. The TAXINOMISIS Project will serve as a platform for the development of new computer tools that assess plaque progression based on radiology images and a lab‐on‐chip with genetic variants that could predict medication response in individual patients.

**Conclusion:**

Results from the TAXINOMISIS study could potentially improve future risk stratification in patients with ACAS to assist personalized evidence‐based treatment decision‐making.

**Strengths and limitations of this study**

- This study will lead to the discovery of new biomarkers by investigating potential underlying pathobiological processes and combining existing data from European biobanks and cohort studies.
- Promising circulating biomarkers will be explored for carotid artery stenosis patients in the same biobanks.
- This is the first study that combines known and newly identified biomarkers of any kind (clinical, imaging and circulating biomarkers) in a multilevel risk stratification model.
- This new multilevel risk stratification model could potentially guide treatment decision for asymptomatic carotid artery stenosis.
- Limitations include the need for external validation of this model in future studies that also take into account procedural risks of revascularization interventions. Thereafter, health technology assessment including feasibility and cost‐effectiveness needs to be performed before implementation of the model in clinical practice.

## 1 INTRODUCTION

Stroke prevention is of utmost importance as it is associated with high mortality rates, accounting for 1.1 million deaths annually in Europe hereby being the third cause of death.^1^ Furthermore, stroke causes high morbidity with long‐term disability and therefore accounts for a high socioeconomic burden. Moderate to severe carotid artery stenosis caused by atherosclerotic plaque formation resulting in progressive narrowing of the lumen is one of the major causes of ischaemic stroke. Rupture or erosion of the plaque surface could lead to cerebrovascular thromboembolism and presentation of cerebral symptoms such as stroke, transient ischaemic attack (TIA) or ocular symptoms such as amaurosis fugax or retinal infarction. Thromboembolisms are believed to account for 80% of all cerebrovascular symptoms while the remaining 20% is deemed to be of haemodynamic origin.^1^, ^2^, ^3^

Current treatment of patients with asymptomatic carotid artery stenosis (ACAS) consists of best medical treatment (BMT): including antiplatelet, antihypertensive and statin therapy, risk factor control and lifestyle coaching.^1^ If indicated, additional intervention with either surgery by carotid endarterectomy (CEA) or endovascular intervention (CAS, carotid artery stenting) may be considered. However, the criteria for defining a high‐risk ACAS that necessitates revascularization are still not supported by high‐level evidence according to the latest European Society for Vascular Surgery (ESVS) guidelines.^1^ Furthermore, as CEA and CAS are associated with a 30‐day stroke/death rate of 1.7%‐3.1%,^4^ accurate patient risk stratification regarding future stroke risk is important for weighing up the indication for intervention.

For symptomatic patients, defined as those that presented with ipsilateral cerebrovascular symptoms in the previous six months, CEA and CAS have been proven to be effective in significant reduction of future stroke and stroke‐related death.^1^ However, CEA or CAS for asymptomatic carotid artery stenosis (eg those that did not have ipsilateral cerebrovascular symptoms in the previous six months) remains controversial.^1^, ^4^ Previous randomized‐controlled trials (RCTs) suggested a modest benefit of CEA in asymptomatic patients, mainly in men younger than 75‐80 years with ≥ 60% stenosis with an average surgical risk (meaning no risk factors that increase procedural complications).^4^, ^5^, ^6^, ^7^ However, these RCTs are outdated, with patient inclusion up to 1993^5^, ^7^ or at maximum 2003,^6^ when BMT did not consist of statins and antihypertensive therapy and more patients were active smokers. Therefore stroke risks in contemporary ACAS patients are considered to be lower, challenging the benefit of CEA.^4^, ^8^

Previous studies have indicated subgroups of ACAS patients with high risk of stroke under BMT in which revascularization as addition to BMT may be beneficial. Markers for increased stroke risk on BMT include clinical factors (eg contralateral TIA or stroke^9^, ^10^, ^11^), cerebral imaging characteristics (eg silent infarction,^12^ impaired cerebrovascular reserve^13^) as well as plaque characteristics on either duplex ultrasound (eg stenosis progression,^9^, ^14^ juxtaluminal black area,^15^ plaque echolucency^10^, ^16^ or plaque area^10^) or magnetic resonance angiography (MRA) (intraplaque haemorrhage^17^, ^18^), as proposed in the latest ESVS guideline (2017).^1^ However, supporting evidence for these markers is based on small studies or non‐prespecified subgroup analyses with BMT that is not analogous to current BMT. Furthermore, follow‐up data regarding medication are often lacking and are thus not taken into account.^11^, ^19^ More importantly, while combination of these markers could improve discriminative predicted ability for risk stratification,^10^, ^15^ thus far no studies have investigated the combination of all these high stroke risk markers. Furthermore, biobank initiatives across Europe,^20^, ^21^, ^22^, ^23^, ^24^ analysing blood samples and atherosclerotic plaque tissue, have identified potential circulating biomarkers which are associated with cardiovascular and cerebrovascular risk.^25^ However, current guidelines do not take these into account in advising patient risk stratification and treatment decision. Therefore, a prospective study in ACAS under current BMT is warranted to evaluate all potential predictors (clinical, imaging characteristics and circulating biomarkers) combined in a risk stratification algorithm for stroke.

In addition to the change in pharmaceutical treatment and lifestyle management, recent reports also reveal a paradigm shift in the pathobiological concepts of the atherosclerotic plaque underlying the development of a major cardiovascular event. New pathobiological insights challenging the concept of the vulnerable plaque as sole underlying mechanism of symptomatic plaques have become available.^26^ Vulnerable plaques are characterized by a thin fibrous cap, a large necrotic core and abundant presence of inflammatory cells which have been shown to be the culprit lesions that rupture in fatal myocardial infarction and stroke.^3^ Studies have suggested that from all plaques with a vulnerable phenotype, only a very small proportion (estimated at < 5%) will rupture, whereas the remaining ones do not cause clinical events.^27^, ^28^ Although vulnerable plaques are considered responsible for the majority of symptomatic patients, an increasing proportion of acute cerebrovascular events seems to originate from phenotypically stable plaques that lack these vulnerable characteristics with significant proteoglycan or glycosaminoglycan accumulation and higher collagen levels.^3^, ^26^, ^29^ Embolism originating from phenotypically stable plaque is probably caused by mechanisms of plaque erosion, yet such mechanisms are not fully understood. Conversely, it has been demonstrated that vulnerable plaques can lose their vulnerable characteristics, while stable plaques can become vulnerable over time depending on exogenous factors, such as medical treatment and lifestyle.^30^ Indeed, previous research showed a shift in carotid atherosclerotic plaque characteristics over the past decade. From 2002 to 2015, 50% reduction of lipid‐rich plaques and intraplaque haemorrhage was observed for both symptomatic and asymptomatic carotid plaques, concomitantly with improvement in cardiovascular risk factor management.^31^

The need for improvement in risk stratification to guide treatment for ACAS and new insights in underlying pathobiology has led to the initiation of the TAXINOMISIS project: ‘A multidisciplinary approach for risk stratification of patients with carotid artery disease’. The Greek word TAXINOMISIS means ‘stratification’. The overall aim of the project is to develop a novel multilevel risk stratification model for contemporary patients with ACAS in a prospective multicentre observational clinical trial (Objective 5, Table 1). This multilevel model will potentially integrate clinical, plaque and cerebral imaging characteristics as well as circulating biomarkers. During earlier phases of the TAXINOMISIS Project, new potential biomarkers and risk stratification tools will be explored (Objectives 1‐4, Table 1). This present report aims to describe the rationale and summarized objectives of the European TAXINOMISIS Project.

**TABLE 1:** Summary of the main objectives of the TAXINOMISIS Project

Objective 1	Exploring new risk and susceptibility factors for symptomatic carotid disease ‐ Unravel pathobiological mechanisms underlying symptomatic plaques by characterizing global gene expression profiles and cellular composition of symptomatic and asymptomatic atherosclerotic carotid lesions. ‐ Assess the predictive ability of circulating biomarkers for carotid artery stenosis patients (plasma ceramides and extracellular vesicles)
Objective 2	Integration of data from longitudinal cohort studies and biobanks (including clinical data, omics data, plaque and circulating biomarkers) to dissect mechanisms mediating symptomatic carotid artery disease for identification of susceptibility and protection factors by machine learning techniques
Objective 3	Development of novel computer tools for MRI and duplex ultrasound to predict plaque growth. These tools will be validated in Objective 5.
Objective 4	Development of a lab‐on‐chip technology incorporating SNPs related to medical therapy effectiveness and resistance to guide personalized medical treatment. Validation will be performed in Objective 5.
Objective 5	Development of the multilevel risk stratification model in an observational multicentre clinical trial including clinical, plaque and brain imaging characteristics and circulating biomarkers. This multilevel risk stratification model will include possible predictors from Objective 1 and 2 and known predictors from literature to evaluate the best combination of predictors for the risk stratification model
Objective 6	To perform cost‐effective analyses of the multilevel risk stratification model, plaque growth computer tools and lab‐on chip device

## 2 METHODS AND ANALYSIS

**FIGURE 1:**
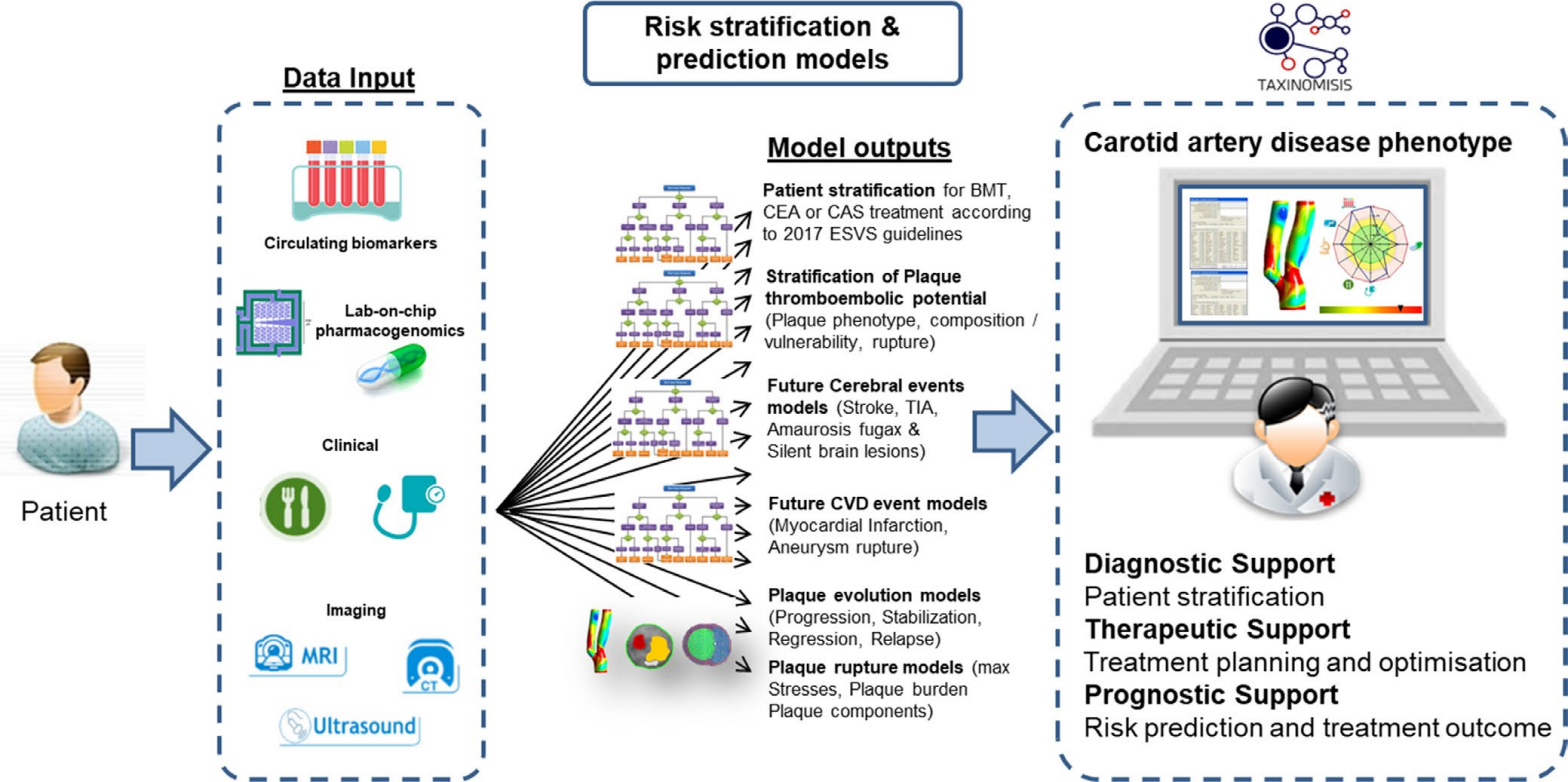
Concept of the TAXINOMISIS Project

The overall aim of the TAXINOMISIS project is to provide a novel multilevel risk stratification model for stroke in patients with ACAS. The main objectives of the project are stated in Table 1 and illustrated in Figure 1. First, underlying pathobiology of symptomatic carotid atherosclerotic plaques will be explored by studying gene expression profiles, cellular composition of plaques in combination with proteomic data (Objective 1). Second, new circulating biomarkers will be investigated with the aim to improve cardiovascular event risk prediction (Objective 1). Third, machine learning techniques will be used to combine available data from existing cohort and biobank studies and explore additional susceptibility and protection factors for cerebrovascular symptoms (Objective 2). Fourth, the final novel multilevel risk stratification model will be developed in a prospective multicentre observational clinical trial (Objective 5). Fifth, new software tools will be developed including computerized models for plaque growth prediction (Objective 3) and lab‐on‐chip pharmacogenomics (Objective 4) for assessment of preventive medication responsiveness. The plaque growth tool and lab‐on‐a‐chip will be validated in the observational clinical trial (Objective 5). Finally, a cost‐effectiveness analyses of the new risk stratification model as well as of novel computer tools will be performed (Objective 6). The project has been initiated at 1 January 2018 and will last until 31 December 2023. A total of 15 research centres and hospitals in 10 different European countries are involved in one or more of the Objectives of the TAXINOMISIS Project (Table 1). The observational clinical study is performed in six major vascular centres (Athens, Barcelona, Belgrade, Genoa, Munich and Utrecht). Reporting of this study conforms to broad EQUATOR guidelines.^32^

### 2.1 Unravelling the pathobiology underlying symptomatic plaques

The gross histopathological classification of a vulnerable plaque is not sufficient to predict all cerebrovascular symptoms.^26^ It is therefore important to further identify molecular mechanisms that contribute to the thrombogenic potential of plaques. In Objective 1, the gene expression profiles (using bulk‐RNA sequencing) and the cellular composition (using mass cytometry, CyTOF, in combination with single‐cell RNA sequencing^33^) of symptomatic and asymptomatic plaques will be studied. This will be combined with available genomic, methylomic and proteomic data in order to identify key biological mechanisms of symptomatic plaques. These new insights could potentially lead to additional plaque or circulating biomarkers that could be candidate markers for the risk stratification model (Objective 5).

### 2.2 New potential circulating biomarkers

Plasma extracellular vesicles (EVs) and ceramides are potential circulating biomarkers for cardiovascular disease risk and will be specifically evaluated for carotid artery stenosis patients in Objective 1. EVs are bilayer membrane particles secreted by every cell of the body into body fluids such as blood. EVs contain lipids, nucleic acids and proteins of the cell of origin; therefore, EVs are often referred to as a ‘liquid biopsy’ of the cell/tissue of origin.^34^ EVs are involved in (patho)physiological processes including intercellular communication, coagulation and inflammation. Content of EVs was previously associated with acute coronary syndrome and metabolic syndrome.^34^, ^35^ Also, four proteins in EVs (Cystatin C, Serpin G1, Serpin F2 and CD14) were associated with an increased risk of future vascular events in patients with manifest cardiovascular disease of any kind.^36^ In Objective 1, the predictive ability of these four EVs proteins for cerebrovascular disease risk will be investigated in patients with carotid artery stenosis.

Plasma ceramides are clinically relevant predictors for cardiovascular events.^37^ Elevated concentrations of circulating ceramides are associated with atherosclerotic plaque formation, ischaemic heart disease, myocardial infarction, hypertension, type 2 diabetes mellitus, insulin resistance and obesity. In particular, specific plasma ceramides Cer(d18:1/16:0), Cer(d18:1/18:0) and Cer(d18:1/24:1) and its ratios with Cer(d18:1/24:0) were associated with cardiovascular death in three large coronary artery disease cohorts.^38^ Still, in carotid artery disease the potential predictive ability of plasma ceramides remains unexplored, and therefore, in Objective 1 the predictive ability of these circulating biomarkers for the risk of acute cerebrovascular complications in carotid artery disease will be evaluated. If EVs and/or plasma ceramides showed to be predictive for stroke, these will be incorporated as possible candidates for the final risk stratification model (Objective 5).

### 2.3 Evaluating new biomarker signatures by integration of biobanks and longitudinal cohorts

In Objective 2, available data from longitudinal cohort studies and biobanks (eg Athero‐Express,^22^ Munich Vascular Biobank,^20^ Tampere Vascular Study^23^ and Young Finns Study^24^) will be combined and integrated. Types of available data are clinical, lifestyle, demographics, histopathological plaque data as well as ‘omics’ data (including genomics, methylomics, transcriptomics and proteomics) and blood and serum biomarkers (proteins and lipids). Furthermore, data from Objective 1, also derived from the same patients in these biobanks, will be incorporated.^22^, ^23^, ^24^ Using machine learning techniques and mathematical models (eg data mining, cluster analysis, latent variable models and multivariate data analysis such as partial list squares), susceptibility and protection factors as well as molecular or biomarker signatures for cerebrovascular symptoms can be investigated. These could potentially be candidate predictors for the novel risk stratification model (Objective 5).

### 2.4 Computational models for plaque growth

The interest in advanced computational models as tools to predict plaque development and plaque growth has fastly developed.^39^, ^40^, ^41^, ^42^, ^43^ Previously, 3D‐reconstruction tools were developed using patient radiology imaging (MRA for carotid arteries^42^ or computed tomography angiography (CTA) for coronary arteries^39^) to analyse atherosclerotic plaque and morphology characteristics. Proof‐of‐concept studies have demonstrated that using mathematical models and simulations of blow flow as well as simulations of different stages contributing to atherosclerotic plaque formation (LDL transport into the vessel wall, recruitment and infiltration of monocytes, oxidation of LDL, differentiation of macrophages into foam cells and proliferation of smooth muscle cells), it was possible to predict regions that are prone to plaque progression.^39^, ^40^, ^41^, ^42^, ^43^ In Objective 3, using retrospective radiology data from TAXINOMISIS participating centres, a computational model for carotid arteries on CTA will be further developed based on the methodology previously used in coronary arteries^39^, ^44^ and the MRA computational model for carotid arteries^40^, ^41^, ^42^ will be further refined for advanced carotid plaque lesions. Finally, both software tools will be validated for prediction of plaque growth in the prospective observational multicentre study (Objective 5) and its clinical utility in risk stratification will be established by relating plaque progression to future cerebrovascular symptoms.

### 2.5 Lab‐on‐chip pharmacogenomics

Clopidogrel and statins are known for interpatient variability in efficacy and side effects. Part of this individual variability could be explained by genetic variations (single‐nucleotide polymorphisms, SNPs) that influence pharmacokinetics and pharmacodynamics of drugs. Well‐known are the CYP2C19*2/ CYP2C19*3 loss‐of‐function alleles for clopidogrel. The prodrug clopidogrel requires biotransformation into its active metabolite by the CYP2C19‐enzyme in the liver. Patients with these loss‐of‐function alleles have lower active metabolite concentrations, therefore diminished platelet inhibition and lower efficacy in preventing cardiovascular complications.^45^ For statins, multiple SNPs are identified that affect its pharmacokinetics, some influence protein transporters that are responsible for uptake or efflux of statins in the liver cell and others influencing the metabolism of statins (influencing CYP‐isoenzymes). Some SNPs lead to decreased statin efficacy whereas others result in higher efficacy inducing toxicity (eg rhabdomyolysis).^46^ In Objective 4, a lab‐on‐chip device will be developed including the important SNPs for statins, clopidogrel and aspirin to assess drug response in the individual patient. A systematic literature search will be performed to identify most clinically relevant SNPs. A proof‐of‐concept lab‐on‐chip device has previously been developed and will be extended for more SNPs.^47^ This lab‐on‐chip could potentially guide personalized treatment by identifying non‐responders and prevent unnecessary side effects. The lab‐on‐chip device will be validated in the observational clinical trial (Objective 5).

### 2.6 Prospective observational multicentre clinical trial

A prospective observational multicentre clinical trial will be performed to develop a new multilevel risk stratification model for cerebrovascular symptoms for patients with ACAS. Participating centres are six major vascular surgery clinics across Europe (Athens, Barcelona, Belgrade, Genoa, Munich and Utrecht). All hospitals have obtained ethical approval by their local ethics committees. The study will be conducted according to the Declaration of Helsinki. All patients must provide written informed consent before study participation. Patients with extracranial ACAS of 50%‐99% are eligible for study participation. Full inclusion and exclusion criteria are displayed in Table 2. According to the 2017 ESVS guideline,^1^ patients are defined as asymptomatic if they have not experienced ipsilateral cerebrovascular symptoms in the past six months. The recruitment period lasts from 31 March 2018 until 31 December 2020. Baseline characteristics of included patients by August 2020 are stated in Table 3.

**TABLE 2:** An overview of the multicentre observational clinical study of TAXINOMISIS

Primary aim	To develop a new multilevel risk stratification model for the three‐year risks of cerebrovascular complications (ipsilateral stroke, TIA, transient or permanent retinal infarction and silent brain ischaemia) in patients with ACAS
Secondary aim	To validate computational models for plaque growth To validate lab‐on‐chip pharmacogenomics for prediction drug response and side effects
Study design	European multicentre observational clinical trial with three years of follow‐up
Inclusion criteria	ACAS of 50%‐99% (in the carotid bifurcation or internal carotid artery) assessed by ultrasound (according to the NASCET criteria).^62^ Patient is 18 y or older Ability of the patient to participate in the follow‐up examinations Personally able and willing to give informed consent
Exclusion criteria	Carotid stenosis due to non‐atherosclerotic causes (eg dissection, fibromuscular dysplasia, carotid aneurysm or post‐irradiation lesions) Tandem carotid lesions (concomitantly stenosis of proximal common carotid artery or brachiocephalic artery) Restenosis after CEA or CAS Increased risk of thromboembolic events (eg congenital or acquired hypercoagulability conditions, active untreated cancer, atrial fibrillation, severe cardiomyopathy with ejection fraction lower than 30%) Intracranial angioma or aneurysms Previous haemorrhagic stroke Planned any major surgery Life expectancy <3 y due to a pre‐existing condition Contra‐indications to best medical therapy (statins, aspirin, clopidogrel) or contra‐indications for MRI examination

**TABLE 3:** Baseline characteristics of included patients

	Total cohort (n = 275)
Demographics	
Male, n (%)	176 (64.0)
Age, mean (SD)	70.0 (8.5)
Medical history	
Ipsilateral carotid stenosis, n(%)	
50%‐70%	133 (48.4)
70%‐99%	142 (51.6)
Contralateral carotid stenosis, n(%)	
0%‐50%	160 (58.2)
50%‐70%	72 (26.2)
70%‐99%	28 (10.2)
100%	15 (5.4)
History of TIA or stroke, n (%)	65 (24.3)
History of stroke, n(%)	37 (13.8)
Previous contralateral CEA or CAS, n(%)	54 (19.6)
History of PAD, n(%)	52 (18.9)
History of CAD, n(%)	71 (27.3)
COPD, n(%)	23 (8.4)
Diabetes, n(%)	81 (29.5)
Risk factors	
Smoking, n(%)	
Never	75 (27.3)
Current	74 (26.9)
Former	126 (45.8)
LDL levels, mmol/L, median [IQR]	2.4 [1.9, 3.0]
HDL levels, mmol/L, median [IQR]	1.3 [1.1, 1.5]
Total cholesterol levels, mmol/L, median [IQR]	4.3 [3.6, 5.0]
Triglycerides levels, mmol/L, median [IQR]	1.3 [1.0, 1.9]
Hypertension, n(%)	218 (83.2)
Glomerular filtration rate, mL/min/1.73 m^2^, median [IQR]	76.7 [60.0, 89.5]
BMI, mean (SD)	26.7 (3.9)
Drug therapy	
Antiplatelets, n(%)	247 (89.8)
Anticoagulants, n(%)	12 (4.4)
Lipid‐lowering drugs, n(%)	238 (86.5)

Note

History of stroke or TIA includes ipsilateral (more than six months prior to inclusion) or contralateral stroke, TIA, amaurosis fugax or retinal infarction. PAD is defined as atherosclerotic lesions in aorta‐iliac or iliofemoral arteries, either treated conservatively or by intervention. CAD is defined as history of angina, myocardial infarction, percutaneous intervention or coronary bypass surgery. Hypertension and diabetes were defined as diagnosed by a medical doctor or use of specific medication. Antiplatelet drug comprises the use of aspirin, dipyridamole or any ADP‐inhibitor. Lipid‐lowering drug use comprises the use of any lipid‐lowering drug.

Abbreviations: n(%), frequencies; SD, standard deviation; IQR, interquartile range; TIA, transient ischaemic attack, CEA, carotid endarterectomy; CAS, carotid artery stenting; PAD, peripheral artery disease; CAD, coronary artery disease; COPD, chronic obstructive pulmonary disease; LDL, Low‐density lipoprotein; HDL, high‐density lipoprotein, BMI, Body mass index.

#### 2.6.1 Candidate predictors for the risk stratification model

The risk stratification model will potentially compromise clinical parameters, imaging (plaque and brain) characteristics and circulating biomarkers. Candidate predictors will be selected based on literature and new biomarkers identified in the earlier phase of the TAXINOMISIS project (Objective 1‐2). Candidate predictors for the model based on previous studies include the following: (a) clinical parameters (demographics such as age, male sex,^48^ medical comorbidities such as worse kidney function,^11^, ^49^ medical history of previous or contralateral stroke or TIA,^11^, ^50^ coronary artery disease, peripheral artery disease and cardiovascular risk factors^50^ such as diabetes, hypertension, smoking, hypercholesterolaemia, high body mass index), (b) radiological characteristics of carotid plaque such as contralateral occlusion,^1^ stenosis progression,^14^ plaque morphology by duplex ultrasound (plaque echolucency,^16^ grayscale median,^10^ juxtaluminal black area,^15^ plaque area^10^), plaque morphology by MRI^17^, ^18^ (intraplaque haemorrhage, lipid‐rich necrotic core, thinning/rupture of the fibrous cap) and (c) circulating biomarkers such as LDL, HDL,^50^ triglycerides, cholesterol, Lp(a),^51^ lipoprotein‐associated phospholipase A2 activity,^52^ CRP,^53^, ^54^ homocysteine,^55^ osteopontine,^23^, ^56^, ^57^ matrix metalloproteinases,^20^ plasma ceramides,^38^ ApoA‐1 auto‐antibodies,^58^ myeloperoxidase,^58^ cystatin C, Serpin F2, Serpin G1 and CD14.^36^

#### 2.6.2 Outcomes

The primary aim is to develop a risk stratification model for cerebrovascular symptoms for patients with ACAS. The primary endpoint is a composite endpoint including cerebrovascular symptoms (stroke, TIA, transient or permanent retinal ischaemia and silent brain ischaemia) during three years of follow‐up. Secondary endpoints include peri‐procedural events, other cardiovascular events (myocardial infarction, abdominal aneurysm rupture or cardiovascular death), plaque progression determined by duplex or MRA and time to revascularization (CEA or CAS) during the three‐year follow‐up.

#### 2.6.3 Sample size

A total of 300 patients will be enrolled across all centres. The prespecified number of inclusion per centre are as follows: Utrecht (UMC), The Netherlands, 50 patients; University of Belgrade (UBEO), Serbia, 100 patients; Technischen Universität München (TUM), Germany: 50 patients; University of Genoa (USMI) Italy, 30 patients; University of Athens (NKUA) Greece, 50 patients; University of Barcelona (FCRB) Spain, 20 patients. A drop‐out of 10% is estimated. Based on previous studies, the combined incidence of TIA, stroke and ocular ischaemia was estimated as 5% per year^10^, ^11^, ^15^ and silent brain ischaemia as 10% per year,^12^, ^59^, ^60^ resulting in a combined event rate of 15% per year. After three‐year follow‐up, 121 patients would reach the primary endpoint. According to the rule of thumb, 10 events per 1 predictor are needed for reliable prediction modelling.^61^ In the final prediction model, a maximum of 12 predictors will be allowed which is considered sufficient and clinically applicable.

#### 2.6.4 Study procedures

Study procedures include collection of clinical data (demographics, medical history and medication use), MRA and duplex ultrasound imaging of both carotid arteries to assess stenosis degree and carotid plaque characteristics, MRI brain for detecting silent brain ischaemia and venous blood sampling for basic laboratory tests (creatinine, lipids, glucose, CRP, homocysteine, Hb and Ht) and circulating biomarkers. These study procedures will be performed at study inclusion and once per year thereafter for a total duration of three years and will be combined with the regular follow‐up visits at the outpatient clinic. Venous blood samples will be stored in freezers of participating centres at −80°C. At the end of the study, circulating biomarkers will be measured centrally to avoid measurement error across centres. During follow‐up, additional clinical data regarding cerebrovascular events and other cardiovascular events (including myocardial infarction, abdominal aneurysm rupture, cardiovascular death or all‐cause mortality) and medication use will be collected. As the study is observational, this study will not interfere with physician's treatment policy for patients with ACAS. When the treating vascular surgeon decides that CEA or CAS is required, patients can still be included in this study. This will be taken into account in data analysis. Biomarker data (brain ischaemia on MRI brain, carotid plaque characteristics on MRA and ultrasound of carotid arteries and circulating biomarkers) collected in the observational study are centrally analysed by independent researchers. Biomarker data are blinded to the treating vascular surgeons to avoid interference with the treatment decision of their ACAS patients.

During CEA, the atherosclerotic plaque will be collected. Immunohistochemical staining for histological plaque characteristics will be performed to determine plaque vulnerability by assessing the amount of lipid core, smooth muscle cells, macrophages, calcification, intraplaque haemorrhage, intraplaque microvessels and collagen. The well‐validated and appreciated standardized protocol of the Athero‐Express Biobank will be used for plaque analyses.^22^

#### 2.6.5 Data management

Data will be collected in predefined electronic case‐report forms (eCRF) through a secured web‐implementation in Open Clinica. Radiology images will be stored in DICOM format and will be collected in a secured online application, specially developed for TAXINOMISIS. Central evaluation of biomarker data (MRI, MRA, ultrasound) and measurements (regarding circulating markers) ensures uniformity of data analyses. Data will be regularly checked for completeness, consistency and quality. Participating centres will be contacted to complement missing or incomplete data. The TAXINOMISIS Project complies with General Data Protection Regulation (GDPR) of the European Union.

#### 2.6.6 Statistical analysis

Cox‐proportional hazard regression modelling will be used for model development. Potential biomarkers that show an independent association with cerebrovascular events will be included. This model will be further reduced using backward stepwise regression analyses based on Akaike information criterion. Calibration of the model will be evaluated through visual inspection of the calibration plot. The discriminative performance of the model will be expressed by Harrell's c‐index and the net‐reclassification index (NRI). Bootstrapping will be used for internal validation to estimate the final model's potential for optimism and overfitting.^61^ Association of biomarkers with secondary endpoints (including peri‐procedural events, plaque progression and other cardiovascular events) will be analysed in sub analyses by Cox‐proportional hazard regression analyses in case of time‐to‐event data or where appropriate by linear or logistic regression analyses.

## 3 DISCUSSION

In the TAXINOMISIS Project, a novel multilevel risk stratification model for cerebrovascular complications of patients with ACAS will be developed. While previous studies have suggested clinical risk factors and imaging and circulating biomarkers for high‐risk patients, this is the first study that combines all these markers in one risk stratification model in patients receiving current standards of BMT. This model could potentially guide personalized treatment decision by identifying those at high risk of future cerebrovascular events that qualify for revascularization procedure in addition to BMT. The main limitation is that this risk stratification model will still need external validation in future studies. It should be noted that this model does not incorporate procedural risks of CEA or CAS, an important factor in determining the indication of revascularization. Procedural risks differ between CEA or CAS, with higher stroke/death rate after CAS, and depend on surgeons' or interventionists’ experience as well as patient characteristics such as anatomy and comorbidities.^1^ Indeed, previous reports based on real‐world registry data have noticed high variability in stroke/death rates across clinics.^1^ Since the number of revascularization procedures in this study is expected to be low as well as the observational study design, this factor could not be incorporated in the model and should require further attention in future studies. Before possible application of a model in clinical practice, health technology assessment including the feasibility and cost‐effectiveness needs to be performed.

## 4 CONCLUSIONS

A new multilevel risk stratification model for patients with ACAS will be developed combining clinical, imaging and circulating biomarkers that have been associated with increased risk of future cerebrovascular symptoms. After external validation, this model aims to guide treatment decision‐making by identifying high‐risk patients that opt for revascularization and prevent unnecessary procedural risks of revascularization in low‐risk patients.

## CONFLICT OF INTEREST

None declared.

## ETHICAL APPROVAL

This work has received funding from the European Union's Horizon 2020 research and innovation programme under grant agreement No 755 320, as part of the TAXINOMISIS project. The observational clinical trial has been approved by the local ethics committees of all participating centres. Results will be presented at international conferences and published in peer‐reviewed journals.

*Ethical approval reference numbers*: Medical ethics committee of the University Medical Centre Utrecht, The Netherlands: Ethics approval acquired with Reference No 18/855; Klinikum rechts der Isar der Technischen Universitat Munchen, Germany: Ethics approval acquired with Reference No: 186/18 S; Faculty of Medicine, University of Belgrade, Serbia: Ethics approval acquired with Reference No: 29/12‐26; National and Kapodistrian University of Athens, Greece: Ethics approval acquired with Reference No: 60/23‐4‐2018; IRCCS Azienda Ospedaliera Universitaria San Martino, Genova, Italy: Ethics approval acquired with Reference No 242/2018; Fundacio privada Clinic per a la recerca Biomedica, Hospital Clinic de Barcelona, Spain: Ethics approval acquired with Reference No HCB/2018/0645.

## Appendix 1 The TAXINOMISIS Consortium

From the Unit of Medical Technology and Intelligent Information Systems, University of Ioannina and Department of Informatics, Ionian University: Themis Exarchos, Panagiotis Siogkas, Vasilis Tsakanikas, Vasiliki Kigka, Xanthi Oustoglou, Vasileios C Pezoulas, Michalis D. Mantzaris, Vassiliki T. Potsika, Dimitrios I. Fotiadis; From the First Propedeutic Department of Surgery, National and Kapodistrian University of Athens, Greece: Konstantinos Filis, Georgios Zografos, Nikolaos Liasis, Georgios Charalampopoulos, Georgios Karagiannis, George Galyfos, Fragiska Sigala; From the Laboratory of Immunobiology, Center for Clinical, Experimental Surgery and Translational Research, Biomedical Research Foundation of the Academy of Athens, Athens, Greece: Lena Siouti, Eleftherios Pavlos, Maria Salagianni, Avraam Hamidie, Kalliopi Thanopoulou, Evangelos Andreakos; From the Department of Vascular Surgery, Faculty of Medicine, University of Thessaly, Thessaly, Greece: Miltiadis Matsagkas; From the Center for Radiology and MRI, Serbian Clinical Centre, Belgrade, Serbia: Ruzica Maksimovic, Marija Jovanovic; From the Clinic for Vascular and Endovascular Surgery, Serbian Clinical Center, Belgrade, Serbia: Stefan Ducic, Petar Zlatanovic, Aleksandra Vujcic, Lazar Davidovic, Igor Koncar, Perica Mutavdzic; From BioIRC, Research and Development Center for Bioengieering, Kragujevac, Serbia: Smiljana Đorović, Branko Arsic, Tijana Djukic, Nenad Filipovic; From the Department of Life Science Technologies, Imec, Leuven, Belgium: Tim Stakenborg, Ahmed Taher, Young Jae Choe, Alejandra Jauregui Uribe, Kirill Zinoviev, Rodrigo Sergio Wiederkehr, Silvia Lenci, Elisabeth Marchal; From the Laboratory of Clinical Chemistry and Hematology, Division Laboratories and Pharmacy, University Medical Centre Utrecht, Utrecht University, Utrecht, the Netherlands: Arjan Boltjes, Gerard Pasterkamp; From the Department of Vascular Surgery, Division of Surgical Specialties, University Medical Centre Utrecht, Utrecht University, Utrecht, the Netherlands: Joost Mekke, Nathalie Timmerman, Gert J. de Borst, Dominique P.V. de Kleijn; From NIVEL, Netherlands Institute for Health Services Research, Utrecht, The Netherlands: Robert Verheij, John Paget, Sytske Wiegersma; From the Clinic of Internal Medicine I, IRCCS Ospedale Policlinico San Martino Genoa – Italian Cardiovascular Network, Genoa Italy: Fabrizio Montecucco; From the Division of Vascular and Endovascular Surgery, IRCCS Ospedale Policlinico San Martino Genoa – Italian Cardiovascular Network, Genoa, Italy: Giovanni Spinella, Maria Bianca Bertolotto, Giovanni Pratesi, Bianca Pane, Domenico Palombo; From Engineering Ingegneria Informatica, Milan, Italy: Luca Longagnani, Roberto Grugni; From the Department of Clinical Chemistry, Faculty of Medicine and Health Technology, Tampere University, Tampere, Finland, the Finnish Cardiovascular Research Center Tampere, Faculty of Medicine and Health Technology, Tampere University, Tampere, Finland and the Department of Clinical Chemistry, Fimlab Laboratories, Tampere, Finland: Pashupati P Mishra, Terho Lehtimäki, Emma Raitoharju, Nina Mononen; From Zora Biosciences Oy, Espoo, Finland and Tampere University, Tampere, Finland: Reijo Laaksonen; From the Clinic and Polyclinic for and Vascular and Endovascular Surgery, Technical University of Munich, Germany: Michael Kallmayer, Joana Goebel, Jaroslav Pelisek, Hans‐Henning Eckstein; From the Kennedy Institute of Rheumatology, Nuffield Department of Orthopaedics, Rheumatology and Musculoskeletal Sciences, University of Oxford, Oxford, UK: Claudia Monaco, Jennifer Cole; From the Vascular Access Unit, Vascular Surgery Division, Department of Cardiovascular Surgery, Hospital Clinic, University of Barcelona, Barcelona, Spain: Vicente Riambau, Victor Obach, Marian Lacentra.
